# High Pressure Extraction as a Green Alternative to the Conventional Sunflower Oil (*Helianthus annuus*) Production Process – Extraction with Pressurized Ethanol in an Intermittent Process and with Supercritical Fluid

**DOI:** 10.1002/gch2.202300335

**Published:** 2024-08-05

**Authors:** Carolina Medeiros Vicentini‐Polette, Beatriz Satie Yamada, Paulo Rodolfo Ramos, Marta Gomes da Silva, Alessandra Lopes de Oliveira

**Affiliations:** ^1^ LTAPPN Departamento de Engenharia de Alimentos Faculdade de Zootecnia e Engenharia de Alimentos (FZEA) Universidade de São Paulo (USP) Av. Duque de Caxias Norte, 225 Pirassununga SP 13635–900 Brazil; ^2^ Instituto de Tecnologia de Alimentos (ITAL) Centro de Ciência e Qualidade de Alimentos Av. Brasil, 2880 Campinas SP 13070–178 Brazil

**Keywords:** green process, pressurized ethanol, sunflower, supercritical carbon dioxide, tocopherol

## Abstract

This research explores green‐technology alternatives to extract vegetable oils as alternatives to hexane, a non‐renewable solvent, focussing on sunflower oil. It compares pressurized liquid extraction (PLE) with ethanol and supercritical fluid extraction (SFE) with CO_2_. Both processes aim to maximize oil yield, tocopherol content (α, β, γ, and δ), fatty acid profile (FA), and triacylglycerol (TAG) composition. Results show that SFE at 32 MPa achieves an 87.58% oil recovery, while PLE at 84 °C achieves 93.93%. PLE with ethanol extracts polar minority compounds along with the oil due to its higher temperature, favoring extraction. The total tocopherol content is 91.17 mg/100 g of oil in optimized SFE conditions, with α‐tocopherol extraction influenced by temperature, γ and δ‐tocopherol by pressure. PLE yields 83.16 mg/100 g of oil in tocopherols influenced less by process variables. The fatty acid (FA) profile do not vary in the oils obtained from different processes or based on the variables within each process, with linoleic and oleic acids being the most abundant. Similarly, triacylglycerols (TAGs) C54:5 and C54:6 are predominant. The optimization of SFE and PLE processes indicates a strong potential for using green solvents in the extraction of tocopherol‐rich sunflower oil.

## Graphical abstract

Consumable vegetable oils are extracted using hexane, a petroleum‐derived, non‐renewable solvent. Green technological alternatives must be studied to replace this conventional technology, which can leave toxic residues in the oil. The optimization of sunflower oil extraction using two methods that employ green solvents, PLE with ethanol and SFE with CO_2_, emerges as a promising alternative for obtaining sunflower oil rich in tocopherol.

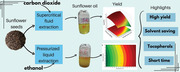

## 1 Introduction

Hexane has been used for decades in the conventional extraction of vegetable oils and fats. After extraction, the crude oil must go through a series of steps before it can be considered to be a refined oil that is suitable for culinary use. These are: filtering, degumming (to remove phospholipids), deacidification, bleaching, and deodorization.^[^ ^1^ ^]^

In the extraction process, which is limited by the solubility of the compounds of interest in solvents, the yield increases linearly with time, especially in the early stages of countercurrent extraction with hexane^[^ ^2^ ^]^ because the extraction occurs by passing the solvent through a fixed bed. Extraction processes also depend on the physical properties of the raw material. In the conventional continuous method of extraction with hexane, the raw material must be pre‐treated by heating (20 min at high temperatures of 90 to 95 °C) and rolling. After extraction, the solvent is recovered from the product and raw material.^[^ ^3^ ^]^

In the conventional oil batch extraction process, extractors are filled with the feed material and then hexane is added. There is a hold time, and the miscella (oil‐solvent mixture) is drained from the system for separation. Currently, this extraction system has been less used due to the long waiting time and the large amount of solvent required.^[^ ^3^ ^]^

The extraction of plant compounds can be made by traditional methods with organic solvents or by supercritical fluid extraction (SFE).^[^ ^4^, ^5^ ^]^ The extraction of vegetable oils and fats by green technologies is the research subject of several authors, and this technology has been implemented in several countries, such as Germany, the United States, France and Japan.

One of the peculiarities of the SFE is related to the characteristics of the supercritical region, where the increase in temperature leads to a decrease in density and an increase in the vapor pressure (*Pv*) of the solutes. This facilitates the migration of these compounds to the solvent,^[^ ^5^, ^6^ ^]^ and the increase in pressure increases the density of the fluid and, consequently, its solubilization power. SFE is considered a green technology because CO_2_ is renewable, and at the end of the process, it is recovered in a gaseous state, preventing its release into the atmosphere. Additionally, it does not leave residues of toxic organic solvents in the extract and matrix.

Supercritical extraction kinetics are normally studied for each different raw material in order to establish the required solvent rate for a suitable extract yield, and consequently, the necessary dynamic extraction time as a function of the flow rate. The study of optimizing extraction processes aims to maximize the quantity of desired components or minimize unwanted ones. The independent variables of the process are examined, and their influence on the dependent variables, such as yield or the content of specific components in the extracts, is statistically analyzed.^[^ ^7^, ^8^, ^9^, ^10^ ^]^

The process of pressurized liquid extraction (PLE) with ethanol as a solvent has currently been characterized as an efficient technique because it reduces extraction times. Its high temperatures (T) decrease the viscosity of the solvent to facilitate its penetration and diffusion without degradation of the solutes, and the high pressure (P) keeps the solvent in the liquid state under T conditions, which would be in the vapor state at atmospheric pressure.^[^ ^11^, ^12^ ^]^ PLE in an intermittent process allows for considerable solvent savings and, when operated with GRAS (Generally Recognized as Safe) and renewable solvents, is classified as a green and sustainable process. In PLE operation, extractions with liquid solvents at temperatures (*T*) above the boiling point are possible through the use of high pressures. Under these conditions, the solvent exhibits greater diffusivity and lower viscosity, facilitating its diffusion through the matrix, increasing mass transfer, and speeding up extractions. In the intermittent process, in addition to the variable temperature (*T*), the volume of the rinsing solvent (VS), the number of extraction cycles (*C*), and the contact time between the matrix and the rinsing solvent in each cycle, referred to as static time (*St*), can be adjusted. At the end of each cycle, during the purging of the extract and the simultaneous entry of the rinsing solvent, the pressure (*P*) and temperature (*T*) of the system remain constant. The volume of rinse solvent (VS) in each cycle is typically expressed as a percentage of the extractor volume, divided by the number of cycles (*C*) used in the process. For example, if the rinse volume is 100% for an extractor with 100 mL capacity and 4 cycles, the rinse volume for each cycle will be 25 mL.^[^ ^13^ ^]^ After the static time (*St*) has elapsed in each cycle (*C*), the solvent‐solute mixture (extract) is purged into the collector, and simultaneously, the solvent (VS) is pumped into the extractor. This process is repeated until the predetermined number of cycles is completed. At the end, the remaining extract is completely purged with nitrogen (N_2_) for 100 seconds.

The extraction from pequi and sacha inchi almonds by PLE using isopropanol as the solvent has been described^[^ ^14^ ^]^ by researchers who evaluated the effects of the static time and temperature for oil extraction yield, free fatty acids (FFAs), total phenolic content (TPC) and β‐sitosterol content. They concluded that static time and temperature positively influenced the yield and TPC, but not the β‐sitosterol content and FFAs.

Further, it has been noted in industry that the use of ethanol in place of hexane for the extraction of sunflower oil has very promising characteristics, and not only does it improve the extraction, it also uses a solvent that is green, GRAS (generally recognized as safe), and renewable.^[^ ^15^ ^]^ Although the use of ethanol also involves a solvent recovery process similar to hexane, the advantage of ethanol is that it is safer for health and renewable.^[^ ^15^ ^]^

As Brazil is the world's largest producer of ethanol, and considering the successful extraction of oil from pequi, sacha inchi,^[^ ^14^ ^]^ and Brazil nut seeds^[^ ^16^ ^]^ with this solvent via PLE in an intermittent process, our research group began studying the use of PLE to obtain soybean oil,^[^ ^17^ ^]^ sunflower oil, and rice bran oil.^[^ ^18^ ^]^ This research has demonstrated that it is possible to achieve high oil extraction yields using pressurized ethanol in short periods of time instead of the traditional hexane process.

Both PLE and SFE offer advantages over the conventional process of extracting vegetable oils with hexane. Hexane is a non‐renewable solvent that is efficient in the extraction process but leaves toxic residues in vegetable oils. In contrast, PLE and SFE use renewable solvents and can be faster. PLE uses smaller amounts of solvents, and SFE leaves no organic solvent residue in the oil. Therefore, in SFE, there is no need to remove the solvent after extraction through distillation, giving it an advantage over both PLE and the conventional hexane extraction method.

Sunflower seed (*Helianthus annuus*) is rich in vitamin E and well known for its content of α‐tocopherol of high biological value. In common sunflower oil, 91 to 97% of the total tocopherol is α‐tocopherol. Seeds with high oil content can reach 1,120 mg of vitamin E/kg of sunflower oil.^[^ ^19^ ^]^ The oil content in seeds can vary for several reasons, including growing conditions such as ambient temperature, soil properties and nutrition, and water availability.^[^ ^20^, ^21^ ^]^

When compared to the conventional process, the extraction of vegetable oils with scCO_2_ allows an efficient extraction of phytosterols and vitamin E together with the oil, and it does so without the residues of organic solvents.^[^ ^22^ ^]^ The efficiency of ethanol in solubilizing tocopherols under atmospheric pressure conditions was demonstrated,^[^ ^23^ ^]^ and the experiment revealed that almost all oil was extracted from sunflower seed pellets within the first ≈17 min, both for T = 50 and at 60 °C. In addition, the authors found that, compared to hexane extraction, ethanol extracted 70% less crystallizable waxes and at least 38% more tocopherols and phospholipids.

This study aimed at presenting a comparative and simplified method for the extraction of sunflower oil enriched in tocopherols by using green technologies, supercritical fluid extraction (SFE), and pressurized ethanol (PLE). The effects of temperature and pressure (SFE) and of temperature, rinse volume, and static time (PLE) were evaluated for the extraction of oil from rolled sunflower seeds, and these provided valuable data about extraction efficiency, oil composition, and tocopherols content.

In addition to presenting two potential technologies for the extraction of vegetable oils used in human food, this research intends to encourage the use of green, “safe for health”, and renewable solvents, when compared to hexane, and further to indicate whether vegetable oils will be more or less enriched in vitamin E depending on the polarity of the solvent (scCO_2_ or ethanol).

## 2 Results and Discussion

### 2.1 Raw Material

The crushed and husked sunflower seeds presented 98.76 ± 0.21% of dry matter (DM); 2.70 ± 0.05% of mineral mater (MM); 23.50 ± 0.26% of crude protein (CP); and 12.88 ± 0.27% of crude fiber (CF). No significant amounts of non‐nitrogen extract (NNE), acid detergent fiber (ADF), ash‐free neutral detergent fiber (NDF), nitrogen in the ADF (N‐ADF), and nitrogen in the NDF (N‐NDF) were found. As for the ethereal extract, the average oil content obtained was 55.07 ± 0.30.

### 2.2 Granulometry

The average diameter of the particles used for extraction was 0.84 mm. This diameter corresponds to the rolled seeds, according to industrial use, and selected at < 30 mm, for partial separation of the husks.

Usually, the extraction yield is inversely proportional to the size of the particles because the smaller the particle, the greater the mass transfer area. For sunflower seeds this behavior was also identified, since the highest oil yield was obtained with 0.23 mm particles (91.7%), while for particles of 0.55 mm, 1.09 mm and 2.18 mm the yields were respectively, 57.2%, 31.9%, and 26.9%.^[^ ^2^ ^]^

In this study, where particles with an average diameter of 0.84 mm were used, the extraction yield in the SFE was 48.23% and in the PLE it was 51.73%, which implies a recovery of 87.58 and 93.93% of seed oil (**Table** **1**).

**Table 1:** Sunflower oil total yield and recovery by supercritical fluid extraction (SFE) (scCO_2_) and by pressurized liquid extraction (PLE) in function of the process variables, temperature (*T*) and pressure (*P*) for SFE and temperature (T) and rinse volume (VS) for PLE in a CCRD.

SFE						
Run	Coded		Variables		Yield [%]	Recovery [%]
	Variables		*T*	*P*		
1	−1	−1	50	20	30.27	54.97
2	−1	1	50	30	47.89	86.96
3	1	−1	70	20	10.73	19.48
4	1	1	70	30	47.32	85.93
5	−1,41	0	46	25	36.92	67.04
6	1,41	0	74	25	24.99	45.38
7	0	−1,41	60	18	12.95	23.52
8	0	1,41	60	32	48.23	87.58
9	0	0	60	25	41.67	75.67
10	0	0	60	25	39.84	72.34
11	0	0	60	25	42.90	77.90

PLE						
Run	Coded		Variables		Yield [%]	Recovery [%]
	Variables		*T*	VS		
1	−1	−1	60	90	42.97	78.03
2	−1	1	60	130	43.03	78.13
3	1	−1	80	90	51.23	93.02
4	1	1	80	130	51.03	92.67
5	−1,41	0	56	110	41.51	75.37
6	1,41	0	84	110	51.73	93.93
7	0	−1,41	70	82	46.65	84.71
8	0	1,41	70	148	48.13	87.39
9	0	0	70	110	46.39	84.24
10	0	0	70	110	47.33	85.94
11	0	0	70	110	47.47	86.20

*T* = Temperature, °C; *P* = Pressure, MPa; VS = Rinse Volume of Solvent, %; Recovery = percentage of the oil obtained in relation to the fat content in the sample (55.07 ± 0.30%).

### 2.3 Supercritical Fluid Extraction (SFE)

This value was very close to that observed in the literature,^[^ ^2^, ^20^, ^25^ ^]^ showing 54.37%, 54.80%, and up to 53.41%; however, lower values are found in the literature, such as 41; 42.2; 45.1; 47.83; and 52%.^[^ ^2^, ^24^, ^25^, ^26^ ^]^ Different values in the oil content of sunflower seeds normally occur due to the variety of the plant, the cultivation conditions and the process conditions employed.

### 2.4 SFE Kinetics

**Figure 1:**
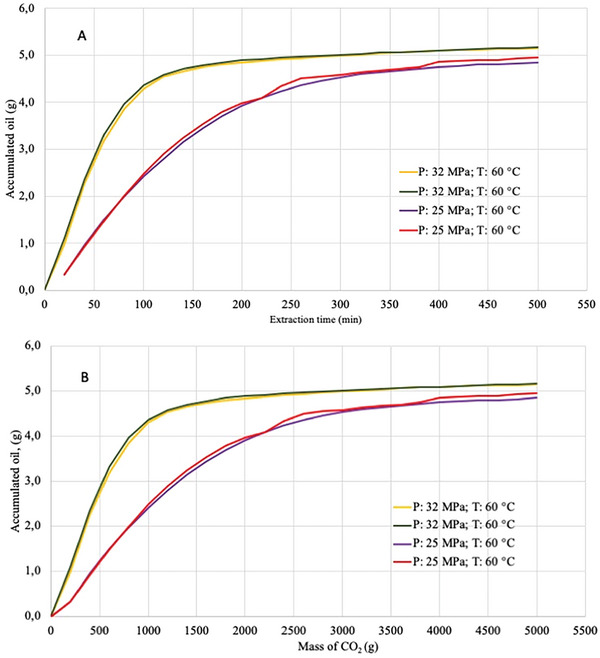
Kinetics of supercritical extraction of sunflower oil, in duplicate, to determine the extraction time when the conditions 25 and 32 MPa, 60 °C and 10 g CO_2_/min are applied; A) kinetics of accumulated oil mass per time (min); B) kinetics of accumulated oil mass per mass of CO_2_.

To choose the extraction time to be performed in the optimization study of the SFE process, the extraction kinetics were evaluated. According to the extraction curve (**Figure** **1**A), the period of 240 min was chosen as viable, where the average yield was 42.2% under the conditions of 25 MPa, 60 °C and 10 g CO_2_/min. In this condition, oil recovery was 76%. Apparently, within 240 min, the increasing extraction rate begins to slow and a plateau starts, which indicates the beginning of extraction controlled by the oil diffusion process through the particles until reaches the surface.^[^ ^20^, ^27^ ^]^ The longer the extraction time, the greater the total yield; however, the processing time must be optimized to be economically viable. Normally, the extraction time used in these processes corresponds to the region of the curve where a constant extraction rate is observed, which occurs between 0 and 150 min at 25 MPa and 60 °C (Figure 1A) from 150 to 240 min, as noted by the period of transition between the constant and the decreasing extraction phases. In the constant extraction rate phase, the external surface of the particles is covered with the solute, which is easily accessible, and convection is the dominant mass transfer mechanism. To confirm that the time chosen was sufficient to extract sunflower oil as much as possible under other conditions, the same kinetics were determined at 32 MPa and 60 °C, which was a higher pressure condition for the same temperature. Under this condition, shorter extraction times (150 min) could be used (Figure 1A).

The total extraction yield provides data to analyze the effects of *T* and *P* in the process and is closely related to the solubility of the solute in the supercritical condition.^[^ ^6^ ^]^ In the extraction kinetics performed at 25 MPa and 60 °C, there was a progressive decrease in the extraction rate after 240 min, in this period. Even though the sunflower seed was in roll format, as used in the industry and which may demonstrate some resistance to mass transfer^[^ ^28^ ^]^ (since the rolled seed has a smaller contact surface than ground materials), 76% oil recovery was achieved (Figure 1A).

Due to the operational cost, the process is usually limited to the region of the curve that concentrates 50 to 70% of extraction yield.^[^ ^29^ ^]^ It must be considered that the relatively short extraction time is one of the characteristics of the best operating conditions.^[^ ^6^ ^]^ The total mass of CO_2_ used in this kinetics was 2.48 kg at the end of 240 min (Figure 1B). Considering that the mass of sunflower seeds packed in the fixed bed extractor for the kinetics study was 10 g and that the mass of CO_2_ used for oil recovery was 2,480 g (Figure 1B), the solvent‐to‐feed ratio (S/F) in this condition was 248. At 32 MPa and 60 °C enough extraction time for a good yield would be 150 min. Under these conditions, the sunflower oil recovery was approximately 88% with a S/F ratio of 150, which implies a considerable reduction in solvent consumption for a higher yield (Figure 1B).

During the study of the extraction behavior by kinetics, the flow of scCO_2_ must be kept constant, as well as pressure and temperature, in order not to let the variation of the solvent mass influence the results obtained.^[^ ^8^ ^]^

The extraction of almond seed oil enriched with tocopherols using scCO_2_ has been described in the literature.^[^ ^10^ ^]^ The experimental variables studied were pressure (*P*) ranging from 35 to 55 MPa, temperature (*T*) ranging from 35 to 50 °C, and scCO_2_ flow rate ranging from 10 to 30 kg h^−1^. The authors concluded that the ideal coextraction condition for oil and tocopherols, including their maximum recovery, was achieved within the first two to three hours of extraction.

### 2.5 Yield of Sunflower Oil Extraction by SFE

**Figure 2:**
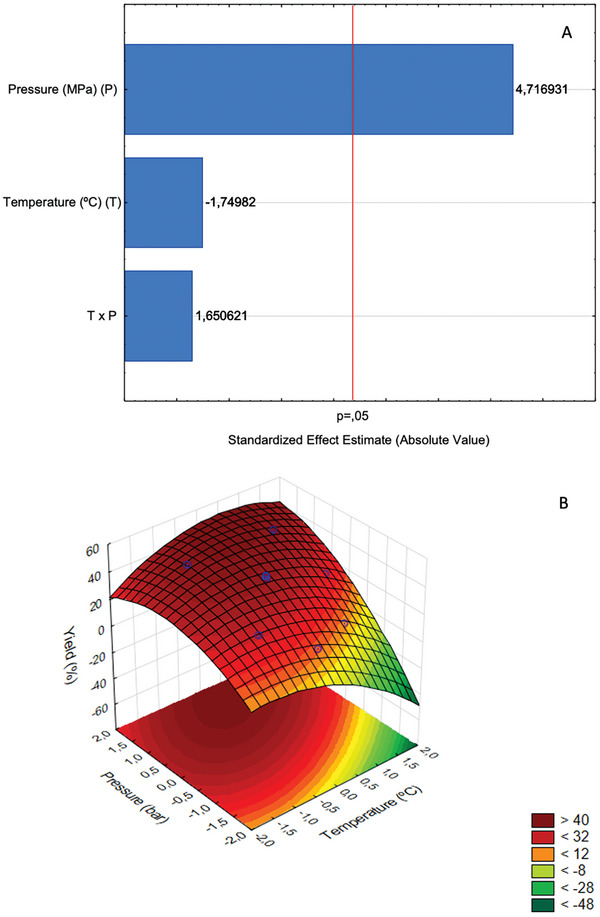
A) Pareto chart for the effects of pressure and temperature on sunflower oil yield by supercritical carbon dioxide extraction. The vertical line indicates the statistical significance bound for the effects. B) Response surface estimated for the yield of the sunflower oil extraction by supercritical carbon dioxide.

The yield of sunflower oil extracted with scCO_2_ varied considerably in the *T* and *P* ranges studied, from 10.73 to 48.23%, which implies an oil recovery of 19.48 and 87.58%, respectively (Table 1). In the statistical analysis of the effects of the process variables in this response, it was found that *P* was the only one that had a significant and positive effect (**Figure** **2**A) at the level of 5% significance.

In the extraction of sunflower oil by scCO_2_, the extraction yield is proportional to the increase in P, which causes an increase in the density of the solvent.^[^ ^24^ ^]^ The extraction rate also increases with increasing scCO_2_ flow.^[^ ^2^ ^]^ Increasing the flow increases the total amount of scCO_2_ used in a given time per fixed amount of seeds. Higher flow rates will imply shorter extraction time. Future research on the economic studies of the process should consider both CO_2_ consumption and extraction time to determine the most economically viable CO_2_ flow.

The yield of sunflower oil extraction by scCO_2_ can be influenced by small variations in *P* and *T* variables, especially at the beginning of the extraction, which is of great importance in optimization for industrial purposes.^[^ ^25^ ^]^ Variations in *P* and *T* change the density of the solvent, and its solubilization power is altered, and consequently, so is the extraction yield. Using scCO_2_, the maximum yield of sunflower oil obtained by Rai et al.^[^ ^20^ ^]^ was about 54.37% when the extraction was performed at 80 °C, 40 MPa, a particle size of 0.75 mm and a solvent flow rate of 10 g/min with 5% ethanol added as co‐solvent. Although this research was a reference for choosing the values of the variables in our process, ethanol was not used as a co‐solvent, and the maximum temperature of our process was 74 °C to avoid the degradation of triacylglycerols, which would increase the acidity of the oil. High temperatures can oxidize the oil, breaking the triacylglycerols into free fatty acids and glycerol.

Fiori^[^ ^9^ ^]^ obtained a yield of up to 39% of sunflower oil using 28 MPa and lower temperature (40 °C). The particle size in this research was also smaller with a mean diameter equal to 0.195 mm for dehulled seeds, and the 0.312 mm for seeds with husk had a yield even lower (23%). These results^[^ ^9^, ^20^ ^]^ show that the use of co‐solvent as well as higher pressure helps to increase yield. In our study, the experimental design used made it possible to identify the best extraction point for sunflower oil with scCO_2_ (48% yield or 87.58% oil recovery) for *P* and *T* (32 MPa and 60 °C) conditions lower than those reported by Rai et al.^[^ ^20^ ^]^ and higher than temperature practiced by Fiori^[^ ^9^ ^]^ (Table 1). Higher pressures generate higher yields while keeping the same temperature and oil quality.

Another study shows the effect of temperature (*T*) on the extraction yield of sunflower oil.^[^ ^8^ ^]^ In this study, the accumulated oil yield is proportional to the increase in temperature, likely due to the increased solubility of triacylglycerols present in the oil, resulting from the volatilization of components at higher temperatures, which facilitates their migration to the solvent.

The solubility of sunflower oil in supercritical carbon dioxide (scCO_2_), with or without ethanol as a co‐solvent, is directly proportional to the extraction rate. Therefore, the solubility can be determined experimentally by the slope of the tangent to the period of constant extraction rate in the extraction kinetics.

Using the yield and coded variables (Table 1) for the statistical analysis of a model to predict the effect of *P* and *T* on sunflower oil yield (*Y*), at least one of the variables was significant (*), *P* (Equation (1)), as already found in the Pareto diagram (Figure 2A). When observing the analysis of variance (ANOVA) and the estimated regression coefficients (R^2^) (Appendix A), which compose the quadratic model, it is observed that all variables showed significance (*) at p < 0.05 (Equation (2)), in addition to an excellent fit.

(1) $$Y_{SFE}=37.23^{*}+13.55^{*}P$$

(2) $$Y_{SFE}=41.47^{*}-4.62^{*}T-4.44^{*}T^{2}+13.01^{*}P-4.62^{*}P^{2}$$

Both linear and quadratic models can be predictive, and this demonstrated the importance of *P* in the supercritical extraction process of sunflower, which could be used in the study of the process in larger scales. In the quadratic model, the quadratic terms of the variables (*T* and *P*) were also significant in predicting the process yield. In addition, when comparing the models, the regression coefficient of the second‐order model (R^2^ = 0.98, adjustment = 0.96) was higher, proving its superior predictive capacity. In both models (Equations (1) and (2)) there was no significant interaction between *P* and *T*. *P* was the most significant factor (p = 0.0065), followed by *T* (p = 0.0076) in the quadratic functions. The highest yield was obtained in the combination of high pressure and moderate temperature, which can also be observed on the response surface generated by the second‐order model (Figure 2B), where it is also observed that extreme temperature values (*T*) (lower and superior) did not favor the process.

The response surface (Figure 2B) resulting from the quadratic model for the extraction yield shows the influence of the factors. The best results can be seen in the region related to the highest *P* employed, where the solvent is found with greater density, carrying the oil. Evaluating the response surface trend, it is clear that higher pressure values could generate higher yields. Experimental confirmation was restricted to equipment limits (35 – 40 MPa). The less optimized points, the condition where the lowest extraction yield was obtained, can be seen in the region with the highest *T*, where the solvent is less dense and, consequently, more volatile (Figure 2B); thus, the scCO_2_ solvent acts in the extraction of rolled sunflower seeds more effectively when it has high density. This behavior is common in the supercritical extraction of vegetable oils. High temperature and high pressure are known to influence the degradation of tocopherols,^[^ ^30^ ^]^ but in this study, relatively mild process conditions were used, which provided a high yield without promoting tocopherol degradation.

Similar results were found by other authors. In the experiment by Rai et al.^[^ ^8^ ^]^ at *T* of 99.85 °C, about 69% of the sunflower oil was extracted before the end of the transition period, while at 59.85 °C, only 54% of the oil was extracted at the end of the same period. In the experiment by Salgin et al.^[^ ^2^ ^]^ and under the conditions they studied, crossover was observed at an overlap between the extraction curves with the *T* when the pressure was 20 and 30 MPa. In this condition for the same *T*, higher *P*’s have higher yields, which indicates the dominance of density over the vapor pressure of the solute (sunflower oil); however, at *P* greater than 30 MPa, the increase in extraction yield was proportional to the increase in applied temperature.^[^ ^2^ ^]^

In the general observation of the referenced researches and of the results obtained in this research, the yield for the extraction of sunflower oil was proportional to the increase of *P*, and was due to the increase of the solubility of the constituents of the sunflower oil, mainly the TAGs^[^ ^2^, ^8^ ^]^ with the increase in fluid density

## 3 Extraction with Pressurized Ethanol (PLE)

### 3.1 Selection of Variables for Obtaining Sunflower Oil by PLE

The significant variables in the extraction process were two, the temperature (*T*) and the volume of solvent (VS) (**Tables** **2** and **3**). Normally, extractions under atmospheric pressure conditions are impacted by both the increase in *T* and the contact time between the matrix and the solvent (*St*), specifically in the PLE of the sunflower extract this time did not significantly interfere, as observed in the preliminary tests in the linear experimental design (Box, Hunter, and Hunter^[^ ^27^ ^]^) (Table 2).

**Table 2:** Yield and recovery observed on the selection the variables analysis by pressurized ethanol extraction of sunflower seed oil, considering the contact time between the solvent and the seeds (*St*), the temperature (*T*), and the rinse solvent volume (VS) in the Box, Hunter, and Hunter design.^[^ ^27^ ^]^

Run	*St* [min]	*T* [°C]	VS [%]	Total Yield [%]	Recovery [%]
1	5	40	120	31.19	56.63
2	9	40	80	29.63	53.80
3	5	60	80	41.10	74.63
4	9	60	120	46.56	84.54
5	7	50	100	39.78	72.23
6	7	50	100	37.02	67.22
7	7	50	100	37.56	68.60

Recovery = in relation to the classical hexane extraction (55.07 ± 0.30%).

**Table 3:** Analysis of variance on variables selection by pressurized ethanol extraction of sunflower seed oil, considering the contact time between the solvent and the seeds (*St*), the temperature (*T*), and the rinse solvent volume (VS) (p ≤ 0.1).

Factor	SS	df	MS	F test	p‐value
(1) *St* (min)	3.80	1	3.80	1.90332	0.261555
(2) *T* (°C)	180.10	1	180.10	90.14607	0.002477
(3) vs (%)	12.32	1	12.32	6.16675	0.089010
Error	6.00	3	2.00		
Total SS	202.21	6			

R_sqr_ = .97036; Adj: .94072; MS Residual = 1.997829. SS = square sum; df = degrees of freedom; MS = mean square.

The difference between conventional extraction of sunflower oil with hexane and PLE is related to pressure and consequently to extraction time. In both processes, an increase in *T* decreases the viscosity of the solvent, which in turn increases its diffusivity through the matrix. Therefore, the diffusion coefficient is proportional to the increase in temperature.

In conventional extraction at atmospheric pressure, the extraction yield increases as a function of the oil content on the seed surface and also the contact time between the solvent and the matrix. The more oil on the surface, the easier it is to extract and the less contact time is required. This time increases from the moment when oil diffusion by the seed becomes dominant in mass transfer.^[^ ^31^ ^]^ In PLE, the high system pressure forces the liquid solvent into the matrix. Due to the high temperature, the liquid solvent has low viscosity and high diffusivity. The pressure acts as a force that facilitates the entry of the solvent through the matrix, thereby speeding up the process. In this study, the extraction time in the PLE varied between 5 and 9 min, a difference of 4 min between the minimum and the maximum, which may be the reason for the non‐interference in the process (Table 2) and therefore not significant in the extraction of sunflower oil. Short contact times (*St*) are very important in the extraction of minor compounds, such as phenolic compounds.^[^ ^32^ ^]^

### 3.2 Yield of Obtaining Sunflower Oil by PLE

In this study, up to 93.93% of the total oil contained in the seed was recovered under conditions of higher temperature (*T*, 84 °C) and a central value of the rinse solvent volume (VS, 110%). The lowest recovery was 75.37% under the conditions of lower *T* (56 °C) and central VS value (110%) (Table 1). In addition, it is expected the PLE presents higher yield of extracts since ethanol, being a polar solvent at high pressure, solubilizes polar compounds together with the oil. Hildebrand's solvency theory can also explain this behavior. In this theory, the square root of the cohesion energy density between molecules expresses a numerical value that indicates the solvency capacity of a specific solvent. This number is called the Hildebrand solubility parameter (δt). Compounds with close to δt usually form mixtures with each other. Thus, solvents that present a δt close to the δt of a solute theoretically result in good extraction efficiency.^[^ ^33^ ^]^ According to Batista et al.^[^ ^34^ ^]^ the total Hansen solubility parameters (δt) for ethanol is 26.52 MPa^1/2^, and for oils used in frying, coconut oil and palm oil, the values are 17.24, 17.40 and 18.31 MPa^1/2^, respectively. Hansen's total solubility for ethanol (δt = 26.52 MPa^1/2^) is greater than that of vegetable oils. Based on the values, it would already be possible to identify that ethanol would not be a good solvent for extracting sunflower oil, but the authors still calculated the relative energy differential (>1) indicating that ethanol would not be a good solvent for oil extraction. Given this, the high oil yields obtained in this research occurred even with the intermittent process at high pressure used in the PLE.

Furthermore, in the conventional extraction of sunflower seeds using Soxhlet, ethanol is able to obtain a higher total yield than hexane and at the same time present a similar content.^[^ ^23^ ^]^ As discussed in the relevant literature, the use of ethanol as a solvent in sunflower oil extraction results in obtaining 70% less crystallizable waxes and at least 38% more tocopherols and phospholipids when compared to hexane extraction. More than 75% of the total sugars are still extracted, mainly raffinose and sucrose. The authors also indicate that after extraction the seeds are an insoluble fraction without the indigestible sugar raffinose.^[^ ^23^, ^34^ ^]^

**Figure 3:**
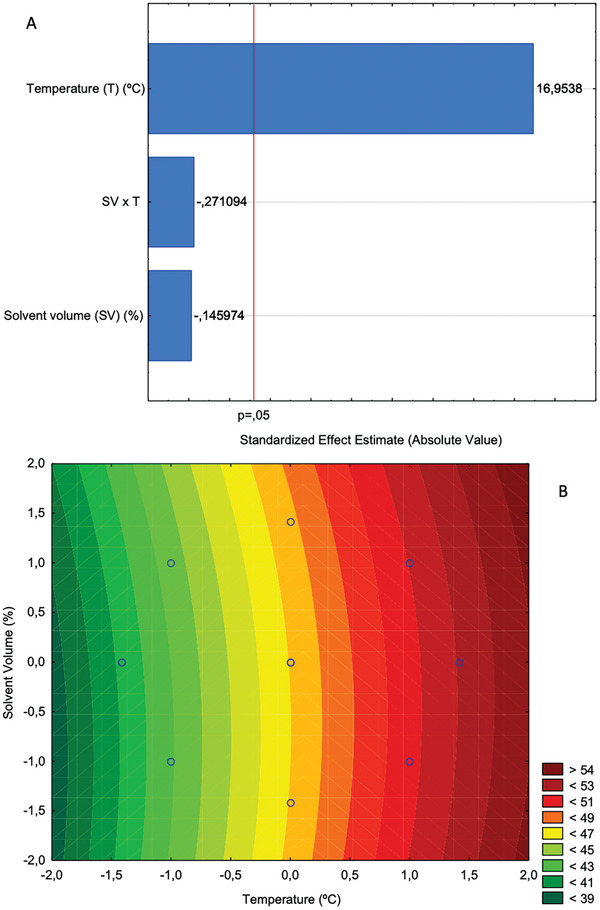
A) Pareto chart for the effects of temperature and rinse volume on sunflower oil yield by pressurized ethanol extraction. The vertical line indicates the statistical significance bound for the effects. B) Response surface estimated for the yield of the sunflower oil extraction by pressurized ethanol.

In the statistical analysis of the yield of the extracts obtained by PLE in function of the process variables (Table 1), specifically in the analysis of the main effects of *T* and VS on the oil yield (*Y*), the only variable that had a significant effect was the temperature (*T*), as can be seen in the Pareto diagram (**Figure** **3**A). The generated models, both linear (Equation (3)) and quadratic (Equation (4)), showed excellent adjustment to the experimental data for the yield of sunflower oil by PLE, with an adjusted coefficient of variation of 0.97 in both models; the model adjustments are shown in the ANOVA for the models in Appendix B.

For both models (Equations (3) and (4)), the only significant coefficient was the linear coefficient for temperature, indicating that the linear model is sufficient to describe the variation in yield with temperature. Figure 3B shows the contour line generated by the quadratic model (Equation (4)), where it is possible to visualize the tendency of increasing the extraction yield with the increase of *T* for the entire range of the rinse solvent volume applied. Although high temperatures can positively influence the oil extraction, they should not be used in this process, as very high temperatures degrade triacylglycerols generating acidic and lower quality oils.

(3) $$Y_{PLE}=47.06^{*}+4.07^{*}T$$

(4) $$Y_{PLE}=47.06^{*}+3.84^{*}T$$

Although in PLE the temperature was the most important variable for a good yield of sunflower oil, in other sunflower oil extraction processes different factors can influence the yield. In a constant temperature process of 50 °C, for example, time was an important factor in the pulsed extraction using ethanol as a solvent. In this process, most of the oil is extracted at the beginning, since the extraction is controlled by the diffusion of the solute to the surface of the particle, regardless of the flow rate. After 95 min of ethanol extraction, 50.3% of the extractable oil was obtained while 41.6% of the oil was obtained by the non‐pulsed method.^[^ ^35^ ^]^ In another process, ethanol was used as a deacidification agent, and the water content added to the solvent influenced the solubility of sunflower oil in ethanol in an inversely proportional way.^[^ ^35^ ^]^

### 3.3 Tocopherol Contents

The total tocopherol content ranged from 15.9 to 91.2 mg per 100 g of sunflower oil in the extracts obtained via SFE and from 40.3 to 83.2 in the oil obtained via PLE‐ethanol (**Table** **4**). Given this, the highest levels obtained by SFE and PLE were 91.2 and 83.2 mg of tocopherols/100 g of sunflower oil, respectively. Of these, 88.0 (SFE) and 77.0 (PLE) mg/100 g correspond to the content of α‐tocopherol (Table 4). The SFE was more selective in the extraction of tocopherols and showed great amplitude in the levels obtained for different conditions of *P* and *T*, while in the PLE the contents were smaller as well as the amplitude.

**Table 4:** Influence of process variables (CCRD) on the dependent variables α, β, γ, δ, and total tocopherols content (mg/100 g) on the sunflower oil extracted by SFE (scCO_2_ extraction), considering the independent variables *T* and *P*, PLE (pressurized ethanol extraction), considering the independent variables *T* and VS.

Run	Tocopherols (SFE)					Tocopherols (PLE)				
	α	β	γ	δ	Total	α	β	γ	δ	Total
1	65.4	5.0	0.7	0.2	71.4	50.0	4.2	1.6	0.2	65.1
2	62.7	0.3	0.3	0.0	63.3	46.2	3.7	1.9	<0.1	50.8
3	29.1	0.8	0.5	0.3	30.66	49.3	3.62	1.87	0.2	54.10
4	22.2	0.4	0.3	0.0	22.89	52.1	3.75	1.47	<0.1	56.64
5	55.8	0.6	0.4	0.2	57.02	77.0	5.36	1.59	0.2	83.16
6	14.9	0.5	0.4	0.1	15.84	36.3	3.02	1.58	<0.1	40.30
7	88.0	1.6	1.2	0.41	91.17	73.5	3.76	1.23	0.2	78.06
8	42.4	0.7	0.5	0.43	44.00	62.9	3.55	1.55	0.2	67.24
9^c^	49.7	0.2	0.4	0.09	49.90	70.3	4.1	0.5	0.2	75.23
10^c^	49.5	0.2	0.4	0.08	49.72	68.9	3.6	0.63	0.19	73.33
11^c^	48.6	0.1	0.4	0.08	48.76	68.3	3.8	0.59	0.19	73.29
C.Al.	40.393.5	<4.5	<3.4	<0.7						
CV%	3.2	4	5.4	4.9	3.5	2.9	3.9	5.4	5.4	2.7

C.Al. = *Codex alimentarius* (FAO, 1999); c = central points; CV% = coefficient of variation.

Commercially Refined Sunflower oil (COM) presented 66.1 (± 0.7) mg/100 g of α‐tocopherol, 2.7 (± 0.1) mg/100 g of β‐tocopherol, 13.0 (± 0.2) mg/100 g of γ‐tocopherol and 2.7 (± 0.4) mg/100 g of δ‐tocopherol, with a total of 84.53 mg/100 g.

### 3.4 Analysis of Tocopherol Content in Sunflower Oil as a Function of SFE Process Variables

In the analysis of the tocopherol content, it is important to highlight that, of the nine tests carried out in this study, only three (33.3%) had an α‐tocopherol content lower than that established by the Codex Alimentarius,^[^ ^36^ ^]^ which stipulates the α‐tocopherol content between 40.3 and 93.5 mg/100 g of sunflower oil. These three conditions (Runs 3, 4 and 6 in Table 4) were those in which higher temperatures were used. This indicates that high temperatures in this process can change the solvent density in order to decrease the scCO_2_ solubilization or even degrade the compounds. As for the other forms of tocopherol, a maximum value normally found in sunflower oils is established, and one of the tests (Run 1, 50 °C, 20 MPa, Table 4) was able to overcome this limit for β‐tocopherol. Refined commercial oil stood out in terms of γ‐(13.02 ± 0.19 mg/100 g) and δ‐tocopherol (2.7 ± 0.08 mg/100 g) levels, being the only one to present values higher than those established by the Codex Alimentarius.^[^ ^36^ ^]^

Compared with commercial and refined versions of sunflower oil, extraction with SFE with central *T* values and lower *P* (Run 7, 60 °C, 18 MPa, Table 4) resulted in higher α‐tocopherol content, while the Run 1 (50 °C, 20 MPa) and 2 (50 °C, 30 MPa) showed values similar to the commercial oil, in addition to surpassing it in the β‐tocopherol content (Table 4). Comparing Run 1 and 2, for the same temperature (50 °C), a higher pressure showed a higher extraction yield (Run 2, Table 1), although not for the tocopherol content in the extracted oil (Table 4). Higher pressures increase the solvent density which allows greater oil extraction (Table 1), but not necessarily for tocopherol extraction. Under conditions of 60 °C and lower pressure (18 MPa), volatilization and migration of minor compounds to the less dense phase occurs due to the higher temperature (Table 4).

When evaluating the influence of the process on the concentration of tocopherols, analysis of the main effects showed the increase of *T*, which negatively influenced the levels of α and δ‐tocopherol (p ≤ 0.05), although this factor was not significant for the levels of β and γ‐tocopherol (p ≤ 0.05). In correlating the solubility of α and δ tocopherol in scCO_2_ (from 40 to 60 °C, and from 9.8 to 33.6 MPa) in equivalent conditions, the solubility of α‐tocopherol was 3 to 4 times lower than that of δ‐tocopherol.^[^ ^37^ ^]^ In our study, we cannot make conclusions about the individual solubility of tocopherols, as their extraction is related to interactions with the lipid components of the oil and interactions between them. There are many compounds competing with the solubilization of scCO_2_.

**Figure 4:**
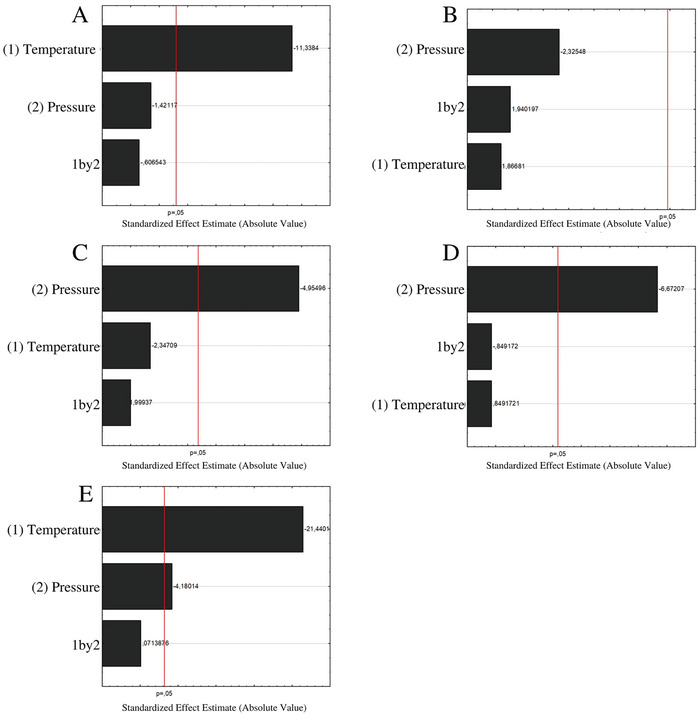
Pareto chart for the effects of pressure and temperature for A) α‐tocopherol, B) β‐tocopherol, C) γ‐tocopherol, D) δ‐tocopherol, and E) total tocopherols in sunflower oil extracted by supercritical carbon dioxide. The vertical line indicates the statistical significance bound for the effects.

In the analysis of the main effects for the yield (Figure 2A), the temperature was not significant; therefore, in the temperature range studied, if the increase in temperature negatively influences the extraction of tocopherols, lower *T*’s can be applied without compromising the yield of the extract. In the statistical analysis of the data, *T* was the most influential variable in obtaining α‐tocopherol (**Figure** **4**A), while *P* was the determinant for γ and δ‐tocopherol (Figure 4C,D). For the total content of tocopherols, both variables were significant (Figure 4E). The interaction between the variables was not significant for any of the tocopherols. Pressure and temperature had a negative influence on the extraction of tocopherols (Figure 4E). This indicates that under optimal extraction conditions, increasing the temperature decreases the density, which is more significant than the potential facilitation of the volatility of these components. The results demonstrated that the pressure facilitated the oil extraction yield (Figure 2B), acting positively and indicating that the higher the solvent density, the better the extraction. This is corroborated in literature, and it is already well known that extraction yield increases with increasing pressure^[^ ^2^, ^20^ ^]^; however, the behavior of SFE in oils and their minor compounds is unique and depends heavily on the raw material.

In using coded variables for the statistical analysis of a model to predict the behavior of *P* and *T* in the levels of tocopherols for sunflower oil obtained by SFE, the ANOVA of the linear model (Figure 4 and Appendix B) indicates that at least one of the variables was significant (*) to obtain α, γ, and δ‐tocopherol, in addition to total tocopherols (Equations (5),(7),(9), and (10)), but the variables are influenced negatively in obtaining the compounds, both individually (*T* and *P*), as well as their interaction (*T* × *P*) for some models.

When analyzing the estimated regression coefficients that compose the quadratic model, it was observed that all parameters (α, β, γ, δ, and total) showed less significance (*) at the level of 5% (Equations (6),(8), and (11)) (R^2^_adj_ = 0.77; 0.70 and 0.81, respectively) when compared to the linear model (R^2^_adj_ = 0.96; 0.94; and 0.98, respectively) as seen in ANOVA (Appendix A). In assessing the influence of process variables on the tocopherol content, linear and quadratic models were generated; however, in the second‐order model none of the quadratic terms was significant (Equations (6),(8), and (11)) except for γ‐tocopherol (Equation (8)). Linear models can be used to predict the behavior of the content of α‐tocopherol (Equation (5)), β‐tocopherol (Equation (7)), and δ‐tocopherol (Equation (9)) in addition to the total of these components (Equation (11)). Considering that the R^2^ for the linear model describing the influence of variables on the γ‐tocopherol content (Equation (7)) was greater than the quadratic model, the linear model can then be used to predict the behavior of all tocopherols as a function of *T* and *P*.

For example, when evaluating the models generated for α‐tocopherol, the adjusted coefficient of determination (R^2^_adj_) was equal to 0.96 for the linear model (Equation (5)) and 0.77 for the quadratic model (Equation (6)). The adjustment for the linear model was higher than the same coefficients of the quadratic model, which was expected, since the quadratic terms of the second‐order model (Equation (6)) are not significant.

(5) $$Y\alpha =45.54^{*}-19.21^{*}T$$

(6) $$Y\alpha =46.51^{*}-16.87^{*}T-9.27^{*}P$$

Considering that the quadratic models generated for the other tocopherols (Equations (6),(8), and (11)) presented the same behavior, i.e., the tocopherol content was influenced by the process variables in the same way as those generated for α‐tocopherol, and the discussion on the behavior of the enrichment of the extracts in these compounds will be carried out based on the linear models (Equations (5),(7),(9), and (10)).

Analyzing the content of the different tocopherols, for β‐tocopherol it was not possible to adjust any predictive model according to process variables because none of the variables showed a significant effect (Figure 4B), which did not occur with the other tocopherols. For γ‐tocopherol, for example, the correlation coefficient was R^2^_adj_ = 0.78 (Equation (7)), but still lower than δ‐tocopherol.

(7) $$Y\gamma =0.44^{*}-0.14^{*}P$$

(8) $$Y\gamma =0.42^{*}-0.19^{*}P+0.17^{*}P^{2}$$

Although it is a minority tocopherol, the linear model proved to be quite predictive for the δ‐tocopherol content, with a determination coefficient of R^2^_adj_ = 0.94 (Equation (9)).

(9) $$Y\delta =0.12^{*}-0.14^{*}P$$

The linear model was also predictive for the total tocopherol content (Ytoc = α + β + γ + δ) (Equation (10)), but in this case, the quadratic model also showed a good fit to the experimental data. The coefficients that were significant in the quadratic model for the extraction yield (Equation (3)) were also for the total tocopherol content (Equation (11)).

(10) $$Ytoc=47.17^{*}-20.27^{*}T-3.95^{*}P$$

(11) $$Ytoc=47.35^{*}-17.45^{*}T-10.32^{*}P$$

**Figure 5:**
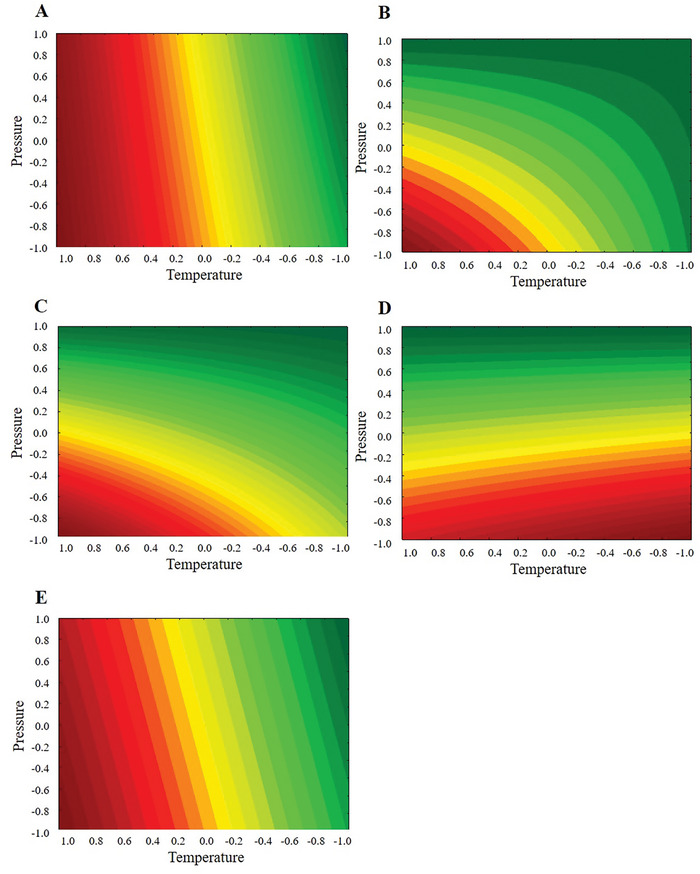
Response surface estimated for the effects of pressure and temperature for A) α‐tocopherol, B) β‐tocopherol, C) γ‐tocopherol, D) δ‐tocopherol, and E) total tocopherols in sunflower oil extracted by supercritical carbon dioxide. The vertical line indicates the statistical significance bound for the effects.

The contour surfaces generated by the linear models show the tendency to obtain higher concentrations of the compounds β, γ, and δ‐tocopherols when lower values of *P* are used (**Figure** **5**B–D); however, when observing the behavior of the levels of α‐tocopherol with *P* and *T*, high values are noted for the entire range of *P* studied, but at lower *T* levels (Figure 5A). Obviously, with α‐tocopherol in greater concentration than the others when assessing the behavior of the concentration of total tocopherols with *P* and *T* (Figure 5E), the same profile is expected.

Extraction with scCO_2_ allows us to fractionate compounds. From the moment that a material rich in a specific component is desired, knowing in which operational condition (*P* vs *T*) this occurs is fundamental for the best solubilization of this compound. For example, if only the content of total tocopherols were analyzed (higher *P*) within the studied range, the concentrations of β, γ, and δ‐tocopherols would probably be lower, so the oil would be poor in these other compounds. Thus, the use of supercritical fluid to obtain sunflower oil with tocopherol content proved to be a viable technology, considering the combination of the variables evaluated.

### 3.5 Analysis of Tocopherol Content in Sunflower Oil as a Function of PLE Process Variables

Compared to the commercial version of sunflower oil, it is noted that extraction with PLE using the lowest *T* and the central values of VS (56 °C, 110% VS, Run 5, Table 4) resulted in a total tocopherol content very close to that observed in the commercial oil (83.2 and 84.6 mg/100 g, respectively); however, when evaluating only α‐tocopherol, it is noted that Run 5 (56 °C, 110% VS) and Run 7 (70 °C, 82% VS), and the central points had a higher content than the commercial one (Table 4). This suggests that, although the commercial oil may contain a higher content of total tocopherols, it is possible to obtain sunflower oil that is even richer in tocopherols and thus present a greater biological value and, consequently, produce a better nutritional quality through the extraction of PLE.

In the extraction of sunflower oil by ethanol, the effective diffusion coefficient (De) for tocopherols is higher than that of phospholipids (3.950 10^−9^ and 2.596 10^−9^ m^2^/s, respectively), independent of the temperature in the analyzed range, in addition to this, both the sugar mass transfer rate and the extraction yield increased with *T* (50 and 60 °C).^[^ ^38^ ^]^ The solubilization of other compounds may contribute to the higher extraction yield.

None of the main effects of the variables *T* and VS on the content of the different tocopherols (Table 4) had significant effects (Appendix C). As a result, it was not possible to generate predictive models that describe the behavior of the content of the different tocopherols in the oil as a function of these PLE variables.

Although both the linear and quadratic models have been shown to be quite predictive for the extraction yield by PLE (R^2^ = 0.99 and R^2^_adj_ = 0.97), the models are not predictive for tocopherols.

Despite the lack of adjustment, only one sample had a lower α‐tocopherol content than that stipulated by Codex Alimentarius^[^ ^36^ ^]^ (Table 4, Run 7, 70 °C, 82% VS). Furthermore, the highest tocopherol content found was 83.2 mg/100 g, which corresponds to the test that obtained the lowest total yield with 75.37% recovery of the total extract. Thus, among the parameters obtained in this study, the most viable condition for obtaining rich sunflower oil with tocopherol by PLE is when using 56 °C for a rinse volume of solvent (VS) of 110% of the volume of the fixed bed extractor (Run 5, Table 4).

Obtaining a sunflower oil that provides both high yield and high tocopherol content is possible by combining the second highest content of α‐tocopherol (73.5 mg/100 g) with a total of oil recovered of approximately 85% with the lowest rinse volume of solvent (82% VS, Run 7, Tables 4 and 7) to provide greater savings and sustainability.

### 3.6 Fatty Acid and Triacylglycerol Profile

Normally, the *P* and *T* conditions used in the supercritical extraction of vegetable oils and animal fats do not change the composition of triacylglycerols and consequently the profile of fatty acids.^[^ ^39^, ^40^ ^]^ In the analysis of the fatty acid profile of sunflower oils obtained by PLE (Runs 1–11, Table 1) and SFE (Run 8, Table 1), no changes in the profile were observed as a function of the process conditions for PLE or between the PLE and SFE process (**Table** **5**).

**Table 5:** Fatty acid profile (FA) of sunflower seed oils obtained by PLE in different conditions of T (°C) and SV (%), SFE at 60 °C and 32 MPa, and COM.

FA (%)		PLE (T °C/VS %)											SFE	COM
		1	2	3	4	5	6	7	8	9	10	11		
		60/90	60/130	80/90	80/130	56/110	84/110	70/82	70/148	70/110	70/110	70/110		
C16:0	Palmitic	6.95	7.21	6.78	6.72	6.94	7.34	6.75	7.19	7.42	6.67	6.73	6.91	7.22
C18:0	Stearic	5.28	5.49	5.03	5.02	5.30	5.66	5.08	5.05	5.51	5.03	5.01	5.03	5.49
C18:1	Oleic	22.58	22.26	22.68	22.6	22.32	24.88	22.16	22.81	24.17	22.59	22.59	22.6	21.65
C18:2	Linoleic	64.43	64.26	64.79	64.91	64.64	61.2	65.25	64.25	62.04	64.96	64.91	64.77	64.73
C22:0	Behenic	0.77	0.79	0.72	0.75	0.8	0.92	0.76	0.7	0.85	0.76	0.76	0.69	0.91

COM = commercial refined oil.

Note that this study obtained values very close to those available in the literature^[^ ^20^, ^24^ ^]^ regardless of the extraction methodology, suggesting that the quality of the oil is maintained when using alternative extraction techniques, including between the oil extracted by scCO_2_ and by propane.^[^ ^24^ ^]^ In this case, pressurized ethanol showed the same selectivity for these components as nonpolar solvents (scCO_2_ and propane).

There is no difference in the fatty acid composition of the oil extracted with scCO_2_ and industrially extracted with hexane (COM) (Table 5) and in the oil a high source of linoleic acid is observed.^[^ ^20^ ^]^ Velez et al.^[^ ^41^ ^]^ also presented the fatty acid composition of sunflower oil with a fatty acid composition of 85% oleic acid (w/w) after non‐catalytic supercritical ethanolysis, 14% palmitic acid (w/w) and 1% of stearic acid (w/w). In this experiment, the linoleic acid (C18:2), being polyunsaturated, suffered greater degradation, hence the difference between the results obtained in our research and in this one. Oleic acid (C18:1) is less susceptible to degradation during the supercritical transesterification reaction of sunflower oil with ethanol and had its concentration increased due to the degradation of linoleic acid. This did not occur in PLE with ethanol (Table 5).

As for the probable composition of TAGs, this study obtained results very close to those obtained by Cuevas et al.^[^ ^42^ ^]^ who also applied a statistic approach for TAG determination with the following exceptions: reporting of the groups 44:1, 46:1, 54:7, not observed in this study, where it was observed as 58:3 and so not reported by the authors. All the other ten groups were compatible (**Table** **6**).

**Table 6:** Triacylglycerols groups (TAGs, %) statistically estimated of sunflower seed oils obtained by PLE in different conditions of T (°C) and VS (%), SFE at 60 °C and 32 MPa, and COM.

Groups	TAG	PLE (T °C/VS %)											SFE	COM
		1	2	3	4	5	6	7	8	9	10	11		
		60 °C, 90%	60 °C, 130%	80 °C, 90%	80 °C, 130%	56 °C, 110%	84 °C, 110%	70 °C, 82%	70 °C, 148%	70 °C, 110%	70 °C, 110%	70 °C, 110%		
50:2	PLP	1.13	1	0.88	0.86	0.92	0.99	0.88	0.98	1.02	0.85	0.87	0.91	1.01
52:2	PLS	3.08	2.63	2.39	2.36	2.49	2.98	2.35	2.56	2.90	2.35	2.36	2.43	2.58
54:2	OOS	1.61	1.36	1.23	1.23	1.3	1.63	1.22	1.25	1.51	1.23	1.22	1.23	1.32
52:3	POL	8.47	7.06	6.78	6.71	6.84	7.69	6.65	7.18	7.64	6.66	6.72	6.89	6.94
54:3	SOL	7.37	6.08	5.82	5.79	5.95	7.03	5.72	5.86	6.66	5.8	5.78	5.79	5.89
58:3	OLB	0.78	0.64	0.6	0.62	0.65	0.80	0.62	0.58	0.72	0.63	0.63	0.56	0.72
52:4	LLP	12.14	10.24	9.73	9.68	9.95	9.51	9.83	10.17	9.86	9.62	9.70	9.91	10.42
54:4	LLS	20.45	16.62	16.54	16.51	16.56	18.00	16.33	16.49	17.5	16.53	16.49	16.46	16.3
58:4	LLB	1.11	0.93	0.85	0.89	0.95	0.99	0.92	0.82	0.93	0.91	0.91	0.81	1.09
54:5	LLO	11.34	27.17	28.19	28.21	27.61	27.62	27.94	27.85	27.55	28.23	28.2	28.06	26.83
54:6	LLL	32.53	26.28	26.98	27.14	26.78	22.76	27.56	26.27	23.69	27.19	27.14	26.94	26.89

P = palmitic acid; L = linoleic acid; S = stearic acid; O = oleic acid; B = behenic acid; COM = refined commercial oil.

## 4 Conclusion

This study shows relevant results regarding the extraction of sunflower oil by two green technologies, supercritical extraction (SFE) with scCO_2_ and extraction with pressurized liquid (PLE) using ethanol as a solvent. These technologies are emerging due to the use of solvents that are safe for health and therefore promising to replace the conventional extraction that uses hexane. Ethanol and scCO_2_ are green solvents and are therefore environmentally friendly extraction techniques.

Both technologies are promising for obtaining sunflower oil with good levels of tocopherol of high biological value using green solvents The yield of sunflower oil extracted with scCO_2_ varied considerably in the temperature and pressure ranges studied, from 19.48 and 87.58% of oil recovery in 240 min (4 h), and with no need of further separation of the solvent, it facilitated the extraction process and provided clean and good quality oil.

PLE provides a facilitated technology with less time of execution and low solvent usage. In about 30 min, the process was able to recover from 75.37 to 93.93% (w:w) of the oil in the seed. Considering the intermittent process, the solvent consumption is considerably lower than conventional methods. The convection stage predominates, indicating that the solvent solubilizes the components on the surface of the particles.

There were, however, differences in the tocopherol content: In oils obtained by SFE, temperature affected α‐tocopherol content, while pressure affected γ‐tocopherol and δ‐tocopherol; therefore, the total tocopherol content was affected by these two variables. In oils obtained by PLE, no variables affected tocopherol content.

Based on the probable composition of TAGs, the products obtained by SFE and PLE proved to be equivalent to the commercial products already available in the Brazilian market. This is considered a good indication, since none of the processes that use green technology and solvents that are safe for health interfere with the lipid composition of the oil while maintaining its characteristics.

In the comparison of SFE with PLE and considering that the yield and the enrichment of the oil in tocopherols was possible for both, although the SFE is a method that does not use organic solvents, and therefore the oil obtained is free from residues of these solvents, the extraction with PLE using ethanol as a solvent (a health‐safe solvent) also has its advantages.

The disadvantage of PLE compared to SFE is the high temperature and solvent removal step after extraction, but polar solvents like ethanol can extract minor compounds in addition to tocopherols that can be used for other purposes.

## 5 Experimental Section

### Materials

SFE was performed using CO_2_ 99.9% (Linde, Sertãozinho, Brazil) as the solvent; PLE was performed using absolute ethanol 99.5% (Dinâmica, Indaiatuba, Brazil) as the solvent. For the tocopherol analysis, *n*‐hexane 98.5% (VWR, Fontenay‐sous‐Bois, France), ethyl acetate 99.8% (Tedia, Fairfield, USA), glacial acetic acid analytical grade 99.7% (Lab Synth, Diadema, Brazil), and a tocopherol set (a convenient bottle pack that contained approximately 50 mg each tocopherols, α, β, γ e δ tocopherols from Calbiochem, Darmstadt, Germany) were used.

The reagents used in the determination of fatty acids were: sodium hydroxide 99% (Êxodo Científica, Hortolândia, Brazil), sodium chloride 99% (Êxodo Científica, Hortolândia, Brazil), sodium sulfate 99% (Synth, Diadema, Brazil), methyl alcohol 99.8% (Dinâmica, Indaiatuba, Brazil), hexane 98.5% (Êxodo Científica, Sumaré, Brazil), *n*‐hexane 98.5% (VWR, Fontenay‐sous‐Bois, France), and boron trifluoride 14% in methanol (Sigma‐Aldrich, St. Louis, MO, USA).

### Raw Material

The seeds originated from sunflower plants (*Helianthus annuus*), variety Altis 99 (Atlântica Sementes) and were kindly donated by Caramuru S/A (Brazil). For characterization, the sunflower seeds were selected, husked, and dried in a forced circulation oven (MA‐035, Marconi, Piracicaba, São Paulo, Brazil) until they reached a constant mass. The seeds were crushed using a household blender (Daily Turbo, Walita‐Philips, Varginha, Brazil), sieved, and stored at −22 °C. The seeds were crushed only for the composition analysis; in the extraction process the seeds were used as they were in the conventional extraction process. In the extraction, sunflower seed flakes formed by Caramuru S/A rolling were used. For analysis of the composition and extraction, the husks were removed, and in the extraction process the flakes seed underwent a partial removal of the husks by hand sieving.

The crushed and husked sunflower seed was characterized by the quantification of dry matter (DM, AOAC method 925.40), mineral matter (MM, AOAC method 950.49), crude protein (CP, AOAC method 950.48), crude fiber (CF, AOAC method 935.53), ethereal extract (EE, AOAC method 948.22), non‐nitrogen extract (NNE), acid detergent fiber (ADF), ash‐free neutral detergent fiber (NDF), nitrogen in the ADF (N‐ADF), and nitrogen in the NDF (N‐NDF).^[^ ^43^ ^]^ Such analyses were performed at the Nutrition Laboratory of the Department of Zootechnics, Faculty of Zootechnics and Food Engineering, University of São Paulo (ZAZ/FZEA/USP), in triplicate, according to the Association of Official Analytical Chemists.^[^ ^44^ ^]^

To measure the diameter of rolled sunflower seed flakes, with the hulls partially removed by hand sieving, 200 g of flakes were deposited onto the Tyler series sieves from 10 to 48 mesh (2 to 0.3 mm), which were placed on a vibrating device (Bertel Indústria Metalúrgica Ltda., Caieiras, Brazil) that shivered for 10 min until the particles were distributed through the sieves according to the aperture size.^[^ ^45^ ^]^ The mass retained in the respective sieves was weighed on a semi‐analytical scale. The average diameter was determined by the retained masses per sieve according to Equation (12) (Sauter mean diameter), where D_i_ = (d_(N‐1)_ + d_i_)/2, with d_i_ representing the aperture diameter of the i‐th sieve (mm), d_(N‐1)_ corresponding to the nominal aperture of the sieve immediately below d_i_ (mm), and x_i_ being the mass fraction of the particles retained in the i‐th sieve.

(12) $$d_{ps}=\left[\frac{1}{\sum_{j\;=\;1}^{n}\left(\frac{x_{i}}{D_{i}}\right)}\right]$$

### Supercritical Extraction

SFE was performed on Thar‐SFC equipment provided by Thar Technologies Inc. (Pittsburgh, PA, USA). The system included high pressure and cosolvent pumps, heat exchangers, an extractor, and a collector. The sample (10.00 g ± 0.01) was placed in a cylindrical stainless‐steel extractor basket (290 cm^3^, with glass beads), which was then inserted into the extraction system.

In preparation for dynamic extraction, CO_2_ was allowed to remain in contact with the matrix for 20 min so that equilibrium conditions of *P* and *T* were achieved and established in the extractor. The pressure was controlled by a backpressure regulator, and the heating was provided by thermal resistances and measured by thermocouples inserted in the fixed bed extractor. From this moment on, constant flow of liquid CO_2_ was measured in the pump (10 g CO_2_/min) during the extraction time, also pre‐established by the study of kinetics. All equipment was automated and operated by the Process Suit for SFE software (Thar, Waters, Milford, MA, USA). The collection of extract was performed continuously during extraction in a flask immersed in an ice bath.

### Kinetics

For this evaluation, 10 g of rolled and sieved seeds were placed in a cylindrical extractor basket. The condition chosen to profile the extraction kinetics (25 MPa and 60 °C) was chosen based on the discussions observed in the literature^[^ ^20^, ^21^, ^22^, ^23^, ^24^, ^25^, ^26^, ^27^, ^28^, ^29^, ^30^, ^31^, ^32^, ^33^, ^34^, ^35^, ^36^, ^37^, ^38^, ^39^, ^40^, ^41^, ^42^, ^43^, ^44^, ^45^, ^46^ ^]^ (**Table** **7**). The condition that provided good performance (preferably with mild T) was chosen in order to preserve the tocopherols, and this was also based on the central point (**Table** **8**) of the experimental design, which was to optimize the extraction process of sunflower oil rich in tocopherols. The kinetic behavior helps in choosing the extraction time, which was confirmed in the optimized experimental condition.

**Table 7:** Optimized conditions of pressure and temperature in the extraction of sunflower oil in supercritical conditions according to literature used in this research.

Pressure (MPa)	Temperature (°C)	Optimized conditions	References
20 – 40	60 – 100	34.5 MPa/80 °C	Rai et al.^[^ ^20^ ^]^
		40 MPa/80 °C	
8 – 25	30 – 60	25 MPa/40 °C	Nimet et al.^[^ ^24^ ^]^
15 – 35	42 – 80	20 MPa/60 °C	Cocero and Calvo^[^ ^46^ ^]^

**Table 8:** Independent variables levels applied to the Central Composite Rotatable Design (CCRD) for supercritical fluid extraction (SFE) of sunflower oil using carbon dioxide.

Variables	Levels				
	−ɑ	−1	0	+1	+ ɑ
Temperature (°C)	46	50	60	70	74
Pressure (MPa)	18	20	25	30	32

The extraction was performed in triplicate, and the extraction time was determined according to the behavior of the material (rolled sunflower seeds) during the SFE, which was scCO_2_ with a flow rate of 10 g CO_2_/min.^[^ ^9^ ^]^ This flow rate and the mass of seeds stored in the fixed bed extractor were chosen due to the limitations of the equipment and also due to the limitations of the collection so that there was no dragging of the extract with the exhaust CO_2_ from the 100 mL flasks changed from time to time. The collections of the extracted sunflower oil were performed periodically every 20 min for 460 min.

### Experimental Design for Supercritical Fluid Extraction of Sunflower Oil

To identify the best conditions for the extraction of sunflower seed oil enriched with tocopherols, a Central Composite Rotatable Design (CCRD) was used and had two independent variables, three central points, and two axial points (−α, + α) (Table 8) that applied 10 g/min of sc‐CO₂ flowrate, 460 min time of extraction, and 10 g of feed material.

The extraction yield and the content of tocopherols were evaluated. Literature and preliminary tests were used to determine the levels for the independent variables, *T* (°C) and *P* (MPa) (Tables 7 and 8 respectively). The central points of the design applied for extraction were selected for kinetics studies as well as the highest yield condition (32 MPa and 60 °C).

### Pressurized Liquid Extraction (PLE)

The oil was also extracted by PLE in a Dionex ASE 150 extractor (Thermo Fisher Scientific, Newington, CT, USA) using 34 mL extraction cells filled with 10 g rolled and sieved sunflower seeds. The PLE used in this experiment performs an intermittent process with extract purge system. The rinse volume of the solvent was chosen according to the capacity of the extraction cell and divided by the number of cycles (*C*) comes into contact with the matrix in each cycle at a set time. After establishing the equilibrium for *P*, which remains constant throughout the extraction, a fraction of the extract was purged and collected for each cycle. *P* was fixed at 10.34 MPa as a limitation of the extractor. During the collection, a new fraction of rinse solvent enters the cell simultaneously. At the last stage, the extract remaining in the extractor was purged with N_2_ to ensure the entire extract was collected.

### Experimental Design for Obtaining Sunflower Oil with Pressurized Ethanol

In the countercurrent solvent extraction industrial process with hexane, the minimum number of contact stages adopted was four,^[^ ^21^ ^]^ and this number was assumed in this study for an intermittent process with pressurized ethanol. The initial conditions (preliminary tests) were stipulated according to the studies of other seeds and similar methods found in the literature,^[^ ^47^ ^]^ and from preliminary tests.

The variables studied in the optimization of the extraction process with pressurized ethanol (99.5%) of sunflower oil enriched with tocopherols were: the static purging time of the solvent (*St*, min), the temperature (*T*, °C) and the rinse volume of solvent (ethanol, VS, %). The number of cycles (*C* = 4) and *P* (10.34 MPa) were set. For the selection of variables, a linear experimental design (Box, Hunter, and Hunter^[^ ^27^ ^]^) was used with three variables and three central points and the extraction yield as a response (Table 3). Significant variables were selected at 90% (p ≤ 0.1).

After selecting variables, a CCRD was applied to two independent variables, three central points and two axial points (‐α, + α) to identify the best conditions for obtaining sunflower seed oil enriched with the tocopherols within the range of this study, which had VS and *T* as independent variables and the extraction yield and tocopherol content as dependent variables. After extraction, the ethanol was recovered in a rotary evaporator (MARCONI, MA‐120, Piracicaba, BR) at 40 °C in a vacuum condition for approximately 30 min (until constant mass).

For the linear and quadratic design, the extraction cell packed with sunflower seeds had its voids filled with ethanol until pressurization (10.34 MPa) and heating (from 56 to 84 °C). Once equilibrium was established, the intermittent process of extracting sunflower oil was started. Four extraction cycles (*C*) and a static time (*St*) of 5 min were used in each cycle. Total time was 20 min. The solvent rinse volume (VS) was variable in the extraction process and was expressed in terms of the extraction cell volume percentage (from 82 to 148%, **Table** **9**). In the intermittent process, the total rinse volume was divided by the number of cycles. For example, for the 34 mL extraction cell, 148% of the volume was 50.32 mL, and this volume in 4 cycles indicates that in each cycle the VS pumped into the system was 12.58 mL. As the solvent was pumped, the extract was collected simultaneously, keeping the *P* and *T* of the system in equilibrium. At the end of the 4 cycles, the extract remaining in the extraction cell was purged with nitrogen. The total extract was collected in a 100 mL glass bottle.

**Table 9:** Levels applied for selection of variables and extraction designs (linear and quadratic) with two and three independent variables for extraction of sunflower oil using pressurized ethanol as a solvent.

Design	Independent variables	−ɑ	−1	0	+1	+ɑ
Selection of variables (2^3^) Box, Hunter, and Hunter design	Contact time [min]	–	5	7	9	–
	Temperature [°C]	–	40	50	60	–
	Rinse Solvent Volume [%]	–	80	100	120	–
Linear and quadratic CCRD	Temperature [°C]	56	60	70	80	84
	Rinse Solvent Volume [%]	82	90	110	130	148

Rinse Volume of Solvent (VS) is expressed in % of extraction cell volume.

### Tocopherol Content

Tocopherol contents were evaluated in all crude sunflower oils and extracts obtained via SFE and PLE and in commercial refined sunflower oil (COM) (Sinhá, Caramuru S/A, Brazil).

The sample (1.0 ± 0.0 g) was diluted to 10 mL with n‐hexane, vortexed for about 1 min, and then filtered through a regenerated cellulose membrane with a 0.45 µm pore size (Pall, Diadema, Brazil). For the quantifications of the β, γ, and δ‐tocopherol, 10 mL of samples were used. For the analysis of α‐tocopherol, only 0.25 mL of sample was used, because it was in higher concentration in the oil. This 0.25 mL was diluted to 10 mL with n‐hexane.

In the analysis, a chromatographic system composed of Rad Pump III (Lab Alliance, Milford, MA, USA) and a fluorescence detector LC 305 (Lab Alliance, Milford, MA, USA) version 3.11 were used. The injector was a Rheodyne type with a 250 µL sampling loop.

The analysis was performed in an isocratic system, using a mobile phase composed of n‐hexane: ethyl acetate: glacial acetic acid (98.8: 0.7: 0.5, v/v/v) and a flow rate of 1.5 mL mi^−1^n. The separation occurred on a 60 Lichrospher Si 60 silica column (Merk, Darmstadt, Germany) (5 µm of film, 125 mm in length, and 4 mm internal diameter).

In monitoring the types of tocopherols, the wavelengths of 294 nm were used for excitation and 326 nm for emission. Quantification was performed by an external standard using a tocopherol standard set. This analysis was performed in duplicate.

### Identification of Fatty Acids (FA) and Probable Triacylglycerols (TAGs) Composition

For the analysis of the fatty acid profile, the crude oil samples were saponified and esterified according to method 969.33^[^ ^44^ ^]^ using hexane as a solvent. After esterification, the samples were diluted to 10% (w/w) in hexane and injected into the chromatograph.

The fatty acid profile of sunflower oils was analyzed by Gas Chromatography coupled to the Mass Spectrometer (GC‐MS), equipped with a Split injector (1:40) (Shimadzu, GCMS‐2010) with an automatic injector (model AOC‐5000) at 250 °C and P = 300 kPa. The capillary column used was the SP 2560 (100 m   × 0.25 mm × 0.2 µm) with helium as the carrier gas at a flow rate of 1.59 mL.min^‐1^.

The oven was started at 100 °C, remaining for 1 min, and was gradually increased (5 °C/min) to 195 °C, and then, with a minor increase (2 °C/min), the temperature was established at 250 °C. The reading occurred between minutes 11 and 60.5, and a mass range between 40 and 350 *m/z*. Peaks with an area ≥ 0.5% were considered in the integration.

The probable content of triacylglycerols (TAGs) in sunflower oils was determined using a statistical analysis.^[^ ^48^ ^]^ TAGs with a frequency ≥ 0.6% were selected.

### Statistical Analysis

The CCRD was planned and analyzed by the Statistica software 13.5.0.17 (Tibco software, Palo Alto, CA, USA) to determine the best conditions for extraction within the range of this study. The results of physical and chemical analysis were subjected to analysis of variance (ANOVA) at a level of significance of p<0.05 using the same software. The estimated TAGs were determined using the MATLAB R2013a Inc. software.

## Conflict of Interest

The authors declare no conflict of interest.

## Supporting information

Supporting Information

## Data Availability

The data that support the findings of this study are available from the corresponding author upon reasonable request.
