# Supplementary material for: High Pressure Extraction as a Green Alternative to the Conventional Sunflower Oil (Helianthus annuus) Production Process – Extraction with Pressurized Ethanol in an Intermittent Process and with Supercritical Fluid
Source: Glob Chall. 2024 Aug 5;8(11):2300335. doi: 10.1002/gch2.202300335 (PMC11557510; doi:10.1002/gch2.202300335)
Supplement: Supplementary file 1 — Supporting Information [file GCH2-8-2300335-s001.docx]

**Supporting Information**

**Appendix A**

**Table A.1.** Regression coefficients, p-value and analysis of variance (ANOVA). Linear and quadratic models for oil extraction yield and tocopherols content of sunflower oil obtained with supercritical fluid extraction (SFE)

| **Linear Model (Ftab = 9.28)** | | | | | | |
| --- | --- | --- | --- | --- | --- | --- |
| Variation Source |  | SS | FD | MS | Fcalc | p-value |
| Regression | Yield | 925.75 | 3 | 308.58 | 9.340 | 0.0495 |
|  | α-tocopherol | 1503.12 | 3 | 501.04 | 56.829 | 0.0038 |
|  | β-tocopherol | 15.04 | 3 | 5.01 | 1.642 | 0.3468 |
|  | λ-tocopherol | 0.112675 | 3 | 0.04 | 8.0651 | 0.0601 |
|  | ∆-tocopherol | 0.078075 | 3 | 0.03 | 31.635 | 0.0090 |
|  | Total tocopherol | 1706.4 | 3 | 568.80 | 93.415 | 0.0018 |
|  |  |  |  |  |  |  |
| Residue | Yield | 99.061 | 3 | 33.02 |  |  |
|  | α-tocopherol | 26.45 | 3 | 8.82 |  |  |
|  | β-tocopherol | 3.05313 | 3 | 1.02 |  |  |
|  | λ-tocopherol | 0.014011 | 3 | 0 |  |  |
|  | ∆-tocopherol | 0.002468 | 3 | 0 |  |  |
|  | Total tocopherol | 18.267 | 3 | 6.09 |  |  |
|  |  |  |  |  |  |  |
| Lack of fit | Yield | 94.319 | 1 |  |  |  |
|  | α-tocopherol | 4.691 | 1 |  |  |  |
|  | β-tocopherol | 2.90086 | 1 |  |  |  |
|  | λ-tocopherol | 0.001811 | 1 |  |  |  |
|  | ∆-tocopherol | 0.002001 | 1 |  |  |  |
|  | Total tocopherol | 0.134 | 1 |  |  |  |
|  |  |  |  |  |  |  |
| Pure error | Yield | 4.7418 | 2 |  |  |  |
|  | α-tocopherol | 21.759 | 2 |  |  |  |
|  | β-tocopherol | 0.15227 | 2 |  |  |  |
|  | λ-tocopherol | 0.0122 | 2 |  |  |  |
|  | ∆-tocopherol | 0.000467 | 2 |  |  |  |
|  | Total tocopherol | 18.133 | 2 |  |  |  |
|  |  |  |  |  |  |  |
| Total | Yield | 1024.8 | 6 |  |  |  |
|  | α-tocopherol | 1529.569 | 6 |  |  |  |
|  | β-tocopherol | 18.0938 | 6 |  |  |  |
|  | λ-tocopherol | 0.126686 | 6 |  |  |  |
|  | ∆-tocopherol | 0.080543 | 6 |  |  |  |
|  | Total tocopherol | 1724.671 | 6 |  |  |  |
|  |  |  |  |  |  |  |
| R²; R² adjusted | Yield | 0.90; 0.81 | |  |  |  |
|  | α-tocopherol | 0.98; 0.96 | |  |  |  |
|  | β-tocopherol | 0.83; 0.66 | |  |  |  |
|  | λ-tocopherol | 0.89; 0.78 | |  |  |  |
|  | ∆-tocopherol | 0.97; 0.94 | |  |  |  |
|  | Total tocopherol | 0.99; 0.98 | |  |  |  |
| **Quadratic Model (Ftab = 5.05)** | | | | | | |
| Variation Source |  | SS | DF | SQM | Fcalc | p-value |
| Regression | Yield | 1794.7 | 5 | 358.95 | 60.027 | 0.0002 |
|  | α-tocopherol | 3850 | 5 | 770 | 7.6539 | 0.0217 |
|  | β-tocopherol | 13.90571 | 5 | 2.78 | 2.7178 | 0.1483 |
|  | λ-tocopherol | 0.573181 | 5 | 0.11 | 5.7992 | 0.0382 |
|  | ∆-tocopherol | 0.137952 | 5 | 0.03 | 1.4529 | 0.3459 |
| *Continues…* | | | | |  |  |
| *…continuation* |  |  | |  |  |  |
|  | Total tocopherol | 4258.203 | 5 | 851.64 | 9.3199 | 0.0143 |
|  |  |  |  |  |  |  |
| Residue | Yield | 29.899 | 5 | 5.9798 |  |  |
|  | α-tocopherol | 503.01 | 5 | 100.6 |  |  |
|  | β-tocopherol | 5.11651 | 5 | 1.02 |  |  |
|  | λ-tocopherol | 0.098837 | 5 | 0.02 |  |  |
|  | ∆-tocopherol | 0.094539 | 5 | 0.02 |  |  |
|  | Total tocopherol | 45.6894 | 5 | 91.38 |  |  |
|  |  |  |  |  |  |  |
| Lack of fit | Yield | 25.157 | 3 |  |  |  |
|  | α-tocopherol | 481.25 | 3 |  |  |  |
|  | β-tocopherol | 4.96424 | 3 |  |  |  |
|  | λ-tocopherol | 0.086637 | 3 |  |  |  |
|  | ∆-tocopherol | 0.094072 | 3 |  |  |  |
|  | Total tocopherol | 438.761 | 3 |  |  |  |
|  |  |  |  |  |  |  |
| Pure error | Yield | 4.742 | 2 |  |  |  |
|  | α-tocopherol | 21.759 | 2 |  |  |  |
|  | β-tocopherol | 0.15227 | 2 |  |  |  |
|  | λ-tocopherol | 0.0122 | 2 |  |  |  |
|  | ∆-tocopherol | 0.000467 | 2 |  |  |  |
|  | Total tocopherol | 18.133 | 2 |  |  |  |
|  |  |  |  |  |  |  |
| Total | Yield | 1824.6 | 10 |  |  |  |
|  | α-tocopherol | 4353.005 | 10 |  |  |  |
|  | β-tocopherol | 19.02222 | 10 |  |  |  |
|  | λ-tocopherol | 0.672018 | 10 |  |  |  |
|  | ∆-tocopherol | 0.232491 | 10 |  |  |  |
|  | Total tocopherol | 4715.097 | 10 |  |  |  |
|  |  |  |  |  |  |  |
| R²; R² adjusted | Yield | 0.98; 0.96 | |  |  |  |
|  | α-tocopherol | 0.88; 0.77 | |  |  |  |
|  |  |  | |  |  |  |
|  | β-tocopherol | 0.73; 0.46 | |  |  |  |
|  | λ-tocopherol | 0.85; 0.70 | |  |  |  |
|  | ∆-tocopherol | 0.59; 0.19 | |  |  |  |
|  | Total tocopherol | 0.90; 0.81 | |  |  |  |

Statistical significance p < 0.05. SS = sum of squares; DF = degress of freedom; MS = mean square.

**Appendix B**

**Table B.1.** Regression coefficients, p-value and analysis of variance (ANOVA). Models for oil extraction yield (linear and quadratic models) and tocopherols content (linear model) of sunflower oil obtained with pressurized liquid extraction (PLE) using ethanol as a solvent

| Linear Model (Ftab = 9,28) | | | | | | |
| --- | --- | --- | --- | --- | --- | --- |
| Variation Source |  | SS | FD | MS | Fcalc | p-value |
| Regression | Yield | 119.046 | 3 | 39.68 | 68.81 | 0.003 |
|  | α-tocopherol | 104.06 | 3 | 186.44 | 1.23 | 0.435 |
|  | β-tocopherol | 0.19 | 3 | 0.06 | 0.21 | 0.886 |
|  | λ-tocopherol | 0.04 | 3 | 0.04 | 0.91 | 0.531 |
|  | ∆-tocopherol | 0.03 | 3 | 0.01 | 1.24 | 0.431 |
|  | Total tocopherol | 111.34 | 3 | 37.11 | 0.24 | 0.863 |
| Residue | Yield | 1.73 | 3 | 0.58 |  |  |
|  | α-tocopherol | 455.28 | 3 | 151.76 |  |  |
|  | β-tocopherol | 0.31 | 3 | 0.10 |  |  |
|  | λ-tocopherol | 0.12 | 3 | 0.04 |  |  |
|  | ∆-tocopherol | 0.02 | 3 | 0.01 |  |  |
|  | Total tocopherol | 459.70 | 3 | 153.23 |  |  |
| Lack of fit | Yield | 1.04 | 1 |  |  |  |
|  | α-tocopherol | 444.50 | 1 |  |  |  |
|  | β-tocopherol | 0.00 | 1 |  |  |  |
|  | λ-tocopherol | 0.05 | 1 |  |  |  |
|  | ∆-tocopherol | 0.02 | 1 |  |  |  |
|  | Total tocopherol | 442.57 | 1 |  |  |  |
| Pure error | Yield | 0.69 | 2 |  |  |  |
|  | α-tocopherol | 10.78 | 2 |  |  |  |
|  | β-tocopherol | 0.31 | 2 |  |  |  |
|  | λ-tocopherol | 0.08 | 2 |  |  |  |
|  | ∆-tocopherol | 0.00 | 2 |  |  |  |
|  | Total tocopherol | 17.13 | 2 |  |  |  |
| Total | Yield | 120.77 | 6 |  |  |  |
|  | α-tocopherol | 559.33 | 6 |  |  |  |
|  | β-tocopherol | 0.50 | 6 |  |  |  |
|  | λ-tocopherol | 0.16 | 6 |  |  |  |
|  | ∆-tocopherol | 0.05 | 6 |  |  |  |
|  | Total tocopherol | 571.03 | 6 |  |  |  |
| R²; R² adjusted | Yield | 0.99; 0.97 | |  |  |  |
|  | α-tocopherol | 0.19; 0.00 | |  |  |  |
|  | β-tocopherol | 0.38; 0.00 | |  |  |  |
|  | λ-tocopherol | 0.24; 0.00 | |  |  |  |
|  | ∆-tocopherol | 0.55; 0.11 | |  |  |  |
|  | Total tocopherol | 0.19; 0.00 | |  |  |  |
|  |  |  |  |  |  |  |
| Quadratic Model (Ftab = 5.05) | | | | | | |
| Variation Source | Yield | SS | DF | MS | Fcalc | p-value |
| Regression |  | 119.05 | 5 | 23.81 | 68.815 | 0.0001 |
| Residue |  | 1.73 | 5 | 0.35 |  |  |
| Lack of fit |  | 1.04 | 3 |  |  |  |
| Pure error |  | 0.69 | 2 |  |  |  |
| Total |  | 120.77 | 10 |  |  |  |
| R²; R² adjusted |  | 0.99; 0.97 | |  |  |  |

Statistical significance p < 0.05. SS = sum of squares; DF = degress of freedom; MS = mean square.

**Appendix C**

**Figure C1.** Pareto chart for the effects of temperature and rinse volume for (A) α-tocopherol, (B) β-tocopherol, (C) γ-tocopherol, (D) δ-tocopherol, and (E) total tocopherols in sunflower oil extracted by pressurized ethanol. The vertical line indicates the statistical significance bound for the effects.
